# Adjuvant chemotherapy for colorectal cancer

**DOI:** 10.1054/bjoc.2001.2081

**Published:** 2001-11

**Authors:** T S Maughan


*British Journal of Cancer* (2001) **85**(10), 1422–1424
© 2001 Cancer Research Campaign
doi: 10.1054/ bjoc.2001.2081, available online at http://www.idealibrary.com on IDEAL

EDITORIAL

### Adjuvant chemotherapy for colorectal cancer

In the last decade, adjuvant chemotherapy for colorectal cancer has become an established component of clinical management. However, considerable controversy still surrounds its application in some patient groups within this population. The original data which changed practice in the USA were based on 2 trials, the original small NCCTG trial (Laurie et al, 1989) and the subsequent confirmatory intergroup study which was updated in 1995 (Moertel et al, 1995) demonstrating increased survival from a dose intensive 12 month 5-fluorouracil plus levamisole adjuvant therapy. This led to the NIH consensus statement in 1991, which mandated adjuvant 5FU and levamisole for Dukes C colon cancer in the USA. As a result, other ongoing trials in the USA were discontinued. Subsequently, data from the pooled analysis of 3 smaller (IMPACT, 1995) and the results of 2 prematurely closed trials (Francini et al, 1994; O'Connell et al, 1997) following the NIH consensus statement have also confirmed the benefits of 5FU in addition to folinic acid over 6 months as effective adjuvant therapy. A meta-analysis of the whole dataset of adjuvant 5FU-based therapy in colorectal cancer estimated the survival benefit to be about 6% (NHS Executive, 1997).

Results from trials comparing surgery plus chemotherapy versus surgery alone remain of particular value in providing further clarification to persisting areas of uncertainty. In this issue, the mature results of the Dutch adjuvant chemotherapy study using 5FU plus levamisole are published (Taal et al, 2001). This study was also prematurely closed in February 1996 in light of the accumulating published data, when 1029 of a planned 2000 patients had been recruited. In the whole group, at nearly 5 years median follow-up, overall survival was improved by the addition of chemotherapy from 55% to 65% ($P$ = 0.007). In node-positive patients the absolute improvement was 12% (44% v 56%). When we discuss the role of adjuvant chemotherapy with our patients, how do we explain to them the degree of benefit that this long and relatively onerous treatment will bring them? How do we help those come to a decision for whom both we, as oncologists and they, as the patients, are uncertain of the relative merits of treatment? What data are in our mind and perhaps in our explanations as we talk to the elderly person in whom other causes of death compete with the risk of recurrence of their carcinoma? Clear data regarding the size of the benefit are important. Is it 6% as the meta-analysis indicates or nearer 12% improvement in survival as the Dutch and IMPACT studies suggest? Given that the meta-analysis included all trials including those with sub-optimal chemotherapy regimens, it is probable that the benefit from now current standard 5FU and folinic acid regimens is nearer 12% than 6%.

The effect of adjuvant chemotherapy for node-negative (stage II) patients remains controversial. In the Dutch study 468 patients were stage II and these showed a similar benefit to the stage III patients with an improved relative survival of 78% versus 70%. These data need to be seen in the light of pooled results from American and European studies in this patient group. In a report of 1565 stage II colon cancer patients from the series of NSABP trials, an equivalent benefit was demonstrated as for the stage III patients with a mortality reduction of 30% for the more active adjuvant therapy (Mamounas et al, 1999). In contrast, 1016 stage II patients entered into 5 separate trials of 5FU plus folinic acid versus observation showed no significant increase in event-free or overall survival at 5 years (Impact B2, 1999). Further data will be forthcoming from the QUASAR trial of over 2000 patients of whom about 80% are node-negative. The current view is that there is little biological difference between node-negative and node-positive cancers and that chemotherapy has a proportionately similar effect in the 2 settings. Because of the smaller overall risk for patients with node-negative cancers, the absolute benefit is smaller. Patients with node-negative disease should be referred to oncologists for discussion about these issues so that the patients may participate in the decision to receive adjuvant therapy. Ideally they should continue to be entered into trials with a no treatment arm, though it is becoming increasingly difficult to obtain informed consent to this randomisation in my experience.

Rectal cancer is an even greater minefield. Because of the concomitant usage of radiotherapy in many patients with rectal cancer, the effect of adjuvant chemotherapy per se is very difficult to determine. In addition, the marked reduction in local recurrence that has occurred with the widespread adoption of total mesorectal excision in some European countries has raised questions about how this will effect benefits from adjuvant therapy. Combined chemotherapy and radiotherapy has been associated with a survival benefit in patients with Dukes B and C rectal cancer, when compared with radiotherapy alone (Krook et al, 1991). One meta-analysis of adjuvant chemotherapy of colorectal cancer (Dube et al, 1997) showed a greater benefit for rectal cancer than for colon cancer (OR for mortality 0.64, 95% CI 0.48–0.85) and estimated the size of benefit to be a 9% increase in survival for rectal cancer patients. NSABP R-02 evaluated chemotherapy with or without post-operative radiotherapy and showed no advantage for survival in adding radiotherapy to adjuvant chemotherapy, but did show an advantage for those randomised to 5FU and folinic acid rather than the alternative schedule (Wolmark et al, 2000). The Dutch study shows that although the effect of adjuvant chemotherapy in rectal cancer was in the same direction as for colon cancer, this did not achieve significance. This negative result must be viewed with caution. Only 299 patients with rectal cancer were entered, half of whom also received radiotherapy. The tests for interaction show no significant heterogeneity, suggesting there was not a qualitatively different phenomenon occurring in the rectal cancer patients. Currently, there is little enthusiasm from patients or oncologists to randomise node-positive rectal cancer patients into surgery-only protocols. Further analysis of all patients entered into randomised trials with a chemotherapy comparison in rectal cancer will clarify this area.

There remain a number of very important questions to be addressed in the adjuvant therapy of colorectal cancer. Treatment regimens are changing. 5FU and levamisole is no longer considered the standard. Equivalent benefits can be obtained with 6 months therapy with 5FU and folinic acid (Haller et al, 1998). The QUASAR trial has confirmed that the addition of levamisole to 5FU and folinic acid brings no further benefit and that low-dose folinic acid is as effective as high-dose (QUASAR, 2000a). In a non-randomised comparison, including nearly 5000 patients that trial also showed that weekly injections rather than 5 times weekly

*Received 14 August 2001*
*Accepted 14 August 2001*

is as effective with much-reduced toxicity (QUASAR, 2000b). Trials currently underway will provide data on the additional benefit of irinotecan and oxaliplatin in combination with 5FU and folinic acid. Usage of oral fluoropyrimidines in the adjuvant setting has already been proven in Japan (Sakamoto et al, 1999) and is under further evaluation in the West. Combinations of oral fluoropyrimidines plus irinotecan or oxaliplatin also need to be evaluated. In the UK, a large trial of cyclo-oxygenase II-selective inhibition, following completion of all standard therapy has just commenced. In due course there will be trials evaluating novel therapies which should have their maximal benefit in situations of minimal residual disease such as anti-angiogenic agents or immunotherapy.

Duration of chemotherapy also needs to be addressed. It has been shown that 12 months therapy of the optimal regimen is no better than 6 months (Haller et al, 1998; Wolmark et al, 1999). In advanced disease, a recent MRC trial showed that 12 weeks of therapy followed by an interval until disease progression resulted in improved quality of life with no detriment to overall survival when compared with continued therapy until progression (Maughan et al, 2001). Could a similar shortened schedule be equally effective in adjuvant therapy? Saini and colleagues reported a trial comparing 12 weeks infusional 5FU versus 6 months 5FU plus folinic acid and reported no detriment in survival (Saini et al, 2000). A shorter schedule with similar effectiveness would have great benefits for patients and the economy.

The major breakthrough will be the ability to define from molecular as well as conventional histopathological criteria, which patients need chemotherapy and which will benefit from a specific combination selected from an increasing array of options. At present data in this area are very confused. Data from a non-randomised retrospective series suggested that all the benefit from adjuvant therapy is due to a major improvement in survival in those patients with tumours expressing high microsatellite instability (Elsaleh et al, 2000). This attractive idea would give an excellent scientific rationale for the difficulty in proving benefit in the rectal subgroup, in whom microsatellite instability occurs in about 1% compared to 15–20% of colonic cancers. However, it has not been substantiated in the large prospective randomised series from the AXIS study which showed no correlation between MSI status and benefit from chemotherapy (Barrett et al, 2001). It also flies in the face of laboratory data which suggest that 5FU is a relatively ineffective drug in these tumours, and that the old alkylating agent CCNU might be the agent of choice (Aguilina et al, 1998).

The identification of poor prognostic factors can be used to identify patients likely to gain more from adjuvant therapy. However, if these factors also result in non-response to chemotherapy, then that supposed benefit will turn out to be illusory. Such a situation appears to be the case for deletions of 18q. This was shown to be a poor prognostic factor by Vogelstein's group, who also suggested that it would therefore be appropriate to select these patients for chemotherapy (Jen et al, 1994). However, one clear message from the AXIS analysis is that loss of 18q and p53 mutation results in a loss of benefit from chemotherapy (Barrett et al, 2001).

The future lies not just in candidate gene analysis but in genetic profiling of tumours to determine the whole range of genetic aberrations present in the individual's tumour. This massive enterprise is now entirely feasible though overwhelmingly expensive using microarray technology. However, there is hope that by measuring the totality of the genomic variation and correlating that with response to the increasing diversity of treatment options, we will be able to identify key factors and pathways which will enable us to select the very best adjuvant therapy for our colorectal cancer patients on an individual basis.

**TS Maughan**

### REFERENCES

Aquilina G, Ceccotti S, Martinelli S, Hampson R and Bignami M (1998) N-(2-chloroethyl)-N'-cyclohexyl-N-nitrosourea sensitivity in mismatch repair-defective human cells. *Cancer Res* **58**: 135–141

Barrett P, Seymour MT, Stenning SP et al (2001) Molecular markers predicting benefit from adjuvant 5-fluorouracil in colon cancer patients. (submitted, Lancet)

Dube S, Heyen F and Jenicek M (1997) Adjuvant chemotherapy in colorectal carcinoma: results of a meta-analysis. *Diseases of the Colon & Rectum* **40**(1): 35–41

Elsaleh H, Joseph D, Grieu F, Zeps N, Spry N and Iacopetta B (2000) Association of tumour site and sex with survival benefit from adjuvant chemotherapy in colorectal cancer. *Lancet* **355**: 1745–1750

Francini G, Petrioli R, Lorenzini L, Mancini S, Armenio S, Tanzini G, Marsili S, Aquino A, Marzocca G and Civitelli S (1994) Folinic acid and 5-fluorouracil as adjuvant chemotherapy in colon cancer. *Gastroenterology* **106(4)**: 899–906

Haller PJ, Catalano JS, Macdonald RJ and Mayer (1998) Eastern cooperative Oncology Group (ECOG);Southwest Oncology Group (SWOG); Cancer and Leukemia Group B (CALGB) Fluorouracil (FU) leucovorin (LV) and levamisole (LEV) adjuvant therapy for colon cancer: Five year final report of Int-0089 D.G. *Proc Am Soc Clin Oncol* **17**; 256 (abstr)

International Multicentre pooled analysis of colon cancer trials (IMPACT) Investigators (1995) Efficacy of adjuvant fluorouracil and folinic acid in colon cancer. *Lancet* **345**: 939–944

International Multicentre Pooled Analysis of B2 Colon Cancer Trials (IMPACT B2) Investigators (1999) Efficacy of adjuvant fluorouracil and folinic acid in B2 colon cancer. *J Clin Oncol* **17**: 1356–1363

Jen J, Kim H, Piantadosi S, Liu ZF, Levitt RC, Sistonen P, Kinzler KW, Vogelstein B and Hamilton SR (1994) Allelic loss of chromosome 18q and prognosis in colorectal cancer. *N Engl J of Med* **331**(**4**): 213–221

Krook JE, Moertel CG, Gunderson LL, Wieand HS, Collins RT, Beart RW, Kubista TP, Poon MA, Meyers WC and Mailliard JA (1991) Effective surgical adjuvant therapy for high-risk rectal carcinoma. *N Engl J Med* **324(11)**: 709–715

Laurie JA, Moertel CG, Fleming TR, Wieand HS, Leigh JE, Rubin J, McCormack GW, Gerstner JB, Krook JE and Mallird J (1989) Surgical adjuvant therapy of large-bowel carcinoma: an evaluation of levamisole and the combination of levamisole and fluorouracil.The North Central Cancer Treatment Group and the Mayo Clinic. *J Clin Oncol* **7(10)**: 1447–1456

Mamounas E, Wieand S, Wolmark N, Bear HD, Atkins JN, Song K, Jones J and Rockette H (1999) Comparative efficacy of adjuvant chemotherapy in patients with Dukes' B versus Dukes' C colon cancer: results from four National surgical Adjuvant Breast and Bowel Project adjuvant studies (C-01, C-02, C-03 and C-04). *J Clin Oncol* **17(5)**: 1349–1355

Maughan TS, James RD, Kerr DJ, Ledermann J, McArdle C, Seymour M, Topham C, Cain D and Stephens RJ (2001) Continuous versus Intermittant chemotherapy for advanced colorectal cancer: Preliminary results of an MRC Cro6b randomised trial. *Proc Am Soc Clin Oncol* **21**: 125a

Moertel CG, Fleming TR, Macdonald JS, Haller DG, Laurie JA, Tangen CM, Ungerleider JS, Emerson WA, Tormey DC and Glick JH (1995) Fluorouracil plus levamisole as effective adjuvant therapy after resection of stage III colon carcinoma: a final report. *Ann Intern Med* **122(5)**: 321–326

NHS Executive (1997) Improving Outcomes in colorectal cancer: the research evidence

O'Connell MJ, Maillard JA, Kahn MJ, Macdonald JS, Haller DG, Mayer RJ and Wieand HS (1997) Controlled trial of fluorouracil and low-dose leucovorin given for six months as post-operative adjuvant therapy for colon cancer. *J Clin Oncol* **15(1)**: 246–250

Quasar collaborative group (2000a) Comparison of fluorouracil with additional levamisole, higher dose folinic acid or both as adjuvant chemotherapy for colorectal cancer: a randomised trial. *Lancet* **355**: 1588–1596

QUASAR colorectal cancer study group (2000b) Adjuvant chemotherapy with 5-fluorouracil, 1-folinic acid and levamisole for patients with colorectal cancer: comparison of weekly versus monthly schedules. *Annals of Oncol* **11**: 947–955

*© 2001 Cancer Research Campaign* 

Saini A, Cunningham D, Norman AR, Hill ME, Tait D, Hickish T, Iveson T, Lofts F, Jodrell D, Ross PJ and Oates JR (2000) Multicentre randomised trial of protracted venous infusion(PVI) 5FU compared to 5FU/folinic acid as adjuvant therapy for colorectal cancer. *Proc Am Soc Clin Oncol* **20**: 2410a

Sakamoto J, Hamada C, Kodaira S, Nakazato H and Ohashi Y (1999) Adjuvant therapy with oral fluoropyrimidines as main chemotherapeutic agents after curative resection for colorectal cancer: individual patient data meta-analysis of randomised trials. *Jpn J Clin Oncol* **29**(**2**): 78–86

Taal BG, van Tinteren H and Zoetmulder FAN on behalf of the NACCP group (2001) Adjuvant 5FU plus levamisole in colonic or rectal cancer: improved survival in stage II and III. *Br J Cancer* xxxxxx

Wolmark N, Rockette H, Mamounas E, Jones J, Wieand S, Wickerham DL, Bear HD, Atkins JN, Dimitrov NV, Glass AG, Fisher ER and Fisher B (1999) Clinical trial to assess the relative efficacy of fluorouracil and leucovorin, fluorouracil and levamisole, and fluorouracil, leucovorin, and levamisole in patients with Dukes' B and C carcinoma of the colon: results from National Surgical Adjuvant Breast and Bowel Project C-04. *J Clin Oncol* **17**(**11**): 3553–3559

Wolmark N, Wieand HS, Hyams DM, Colangelo L, Dimitrov NV, Romond EH, Wexler M, Prager D, Cruz AB Jr, Gordon PH, Petrelli NJ, Deusch M, Mamounas E, Wickerham DL, Fisher ER, Rockette H and Fisher B (2000) Randomized trial of postoperative adjuvant chemotherapy with or without radiotherapy for carcinoma of the rectum: National Surgical Adjuvant Breast and Bowel Project Protocol R-02. *Journal of the National Cancer Institute* **92**(**5**): 388–396

*© 2001 Cancer Research Campaign*

